# 2723. High Mortality and Resistance in Patients with Mucosal Barrier Injury Laboratory-Confirmed Bloodstream Infections (MBI-LCBI)

**DOI:** 10.1093/ofid/ofad500.2334

**Published:** 2023-11-27

**Authors:** Grace Johanna Salazar, Flavio Reyes, Johana Salgado, Margarita Galarza, Amy Peralta

**Affiliations:** Hospital Vozandes Quito, Quito, Pichincha, Ecuador; Hospital Oncologico SOLCA Quito, Quito, Pichincha, Ecuador; Hospital SOLCA Quito, Quito, Pichincha, Ecuador; Hospital de Especialidades Eugenio Espejo, Quito, Pichincha, Ecuador; Centro de Investigación en Enfermedades Infecciosas, México, Distrito Federal, Mexico

## Abstract

**Background:**

Since its definition, MBI-LCBI is a pathology increasingly studied and frequent, however, with the growing threat of multidrug resistance (MDR), it is very important to have pharmacological resources for the treatment and local epidemiology. The economic constraints that many institutions suffer in lower middle-income countries may result in worse outcomes in MBI-LCBI and carbapenem-resistant Enterobacteriaceae (CRE) patients. In Ecuador, only ceftazidime/avibactam is available for the CRE treatment, and it is not considered in the Ecuadorian National formulary (EFN), that means it is not available in public health institutions.

**Methods:**

A retrospective study was conducted in two cancer reference hospitals in Ecuador, from January 2019 to December 2021. All patients with MBI-LCBI according to CDC criteria were included. Demographic, diagnostic, and microbiological isolation data were collected. Strains identification and susceptibility were performed on Vitek 2 system and Kirby Bauer, following CLSI 2022 recommendations and cut-off points. The CRE resistance mechanism was confirmed by PCR. Thirty-day episode mortality was assessed, and compared between susceptible and MDR cases. Data are shown with means, and Chi-Square test was used to evaluate differences between mortality. SPSS version 25 was used.

**Results:**

148 episodes of MBI-LCBI in cancer patients were detected. Figure 1 shows etiology and susceptibity of strains. Sixty one (41.3%) MBI-LCBI causing microorganisms were MDR; 25 (68.8%) were BLEE/AmpC isolates and 19 (31.1%) were CRE. The mortality rate of MBI-LCBI patients was 48.6% (72). There was no difference between deaths from BLEE/AMPc versus susceptible bacteremia (22,2% vs. 27,6% p=0,44), however, mortality from CRE strains was higher compared to susceptible strains (20,8% vs. 2,6% p=0.001) figure 2. The risk of dying with MBI-LCBI with CRE strains was OR: 4.5 (95% CI;1.3-15; p=0.01).

**Figure:**
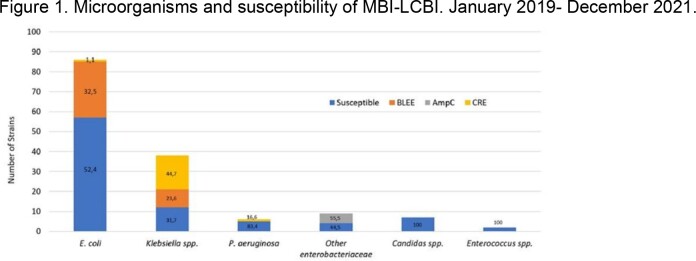

**Figure:**
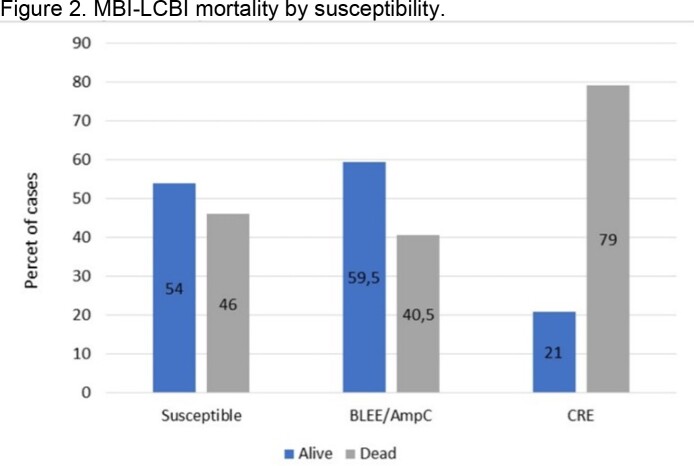

**Conclusion:**

There is a high percentage of resistance and mortality in MBI-LCBI, more notorious when the cause is CRE. This alerts us to the need of further research in this pathology and its appropriate treatment. Ecuadorian health authorities should consider review the EFN to include new active drugs for treatment CRE infections.

**Disclosures:**

**All Authors**: No reported disclosures

